# Awareness of Pleiotropic and Cardioprotective Effect of Statins in Patients with Coronary Artery Disease

**DOI:** 10.1155/2018/8961690

**Published:** 2018-06-07

**Authors:** Bugra Ozkan, Özcan Örsçelik, Hakan Uyar, Mehmet Ballı, Eren Güçer, Onur Aslan, Gülhan Temel, Ahmet Çelik, İsmail Türkay Özcan

**Affiliations:** ^1^Department of Cardiology, Mersin University, Faculty of Medicine, Mersin, Turkey; ^2^Department of Cardiology, Mersin City Hospital, Mersin, Turkey; ^3^Department of Cardiology, Tarsus State Hospital, Mersin, Turkey; ^4^Department of Biostatistics, Mersin University, Faculty of Medicine, Mersin, Turkey

## Abstract

**Background:**

Statins are commonly used in the secondary prevention of coronary artery disease. Studies have shown that the rate of statin use is low among patients with coronary artery disease. In this study, we aimed to investigate the reasons for poor patient compliance with statin treatment.

**Methods:**

A total of 504 patients diagnosed with coronary heart disease were included in the study. Patients were asked 5 questions to assess their level of knowledge about statin therapy.

**Results:**

Among the patients not using statins, 42% stated they did not take the medication because their cholesterol was not high or they did not know they should renew their prescription when they ran out and 35% because they were influenced by news reports in the media suggesting that cholesterol-lowering drugs were harmful. When patients who were aware of the pleiotropic/cardioprotective effects of statins were compared with patients who were not, the more knowledgeable patients had lower noncompliance rate and mean LDL-C level and a higher rate of LDL-C level optimization.

**Conclusion:**

We found that patients who are aware of the pleiotropic effects of statins were more compliant with treatment. We believe that spending more time explaining and emphasizing the mechanisms of action, reason for prescribing, and necessary treatment duration of drugs that patients must use will result in greater compliance and improve patient care. In this way, patients may be less influenced by misinformation presented by the media.

## 1. Introduction

Coronary artery disease (CAD) is a major cause of death and disability in developed countries. Despite a substantial reduction in CAD mortality in recent years, CAD is responsible for approximately 1 in 3 deaths in people over the age of 35 [1]. Numerous studies have demonstrated that across all age groups and both genders, statins reduce mortality and morbidity by both primary and secondary prevention of cardiovascular disease (CVD) and slow the progression of or even reverse atherosclerosis [2, 3]. Statins are among the most studied drugs in the area of CVD prevention and have been incorporated into current treatment guidelines. In the 2016 ESC/EAS Guidelines for the Management of Dyslipidemias, patients with a history of acute coronary syndrome (ACS) or percutaneous coronary interventions (PCI) are considered a very-high-risk group, and initiating cholesterol-lowering medical therapy together with lifestyle changes is recommended for patients in this group with low-density lipoprotein-cholesterol (LDL-C) levels over 70 mg/dL [4]. Evidence regarding the use of statins at both standard and high doses in patients with CAD is not limited to their LDL-C lowering effects. Some of the beneficial effects of statins may be explained by their other “pleiotropic” effects [5].

In contradiction to scientific evidence, reports have appeared in the media in recent years claiming that cholesterol-lowering medications are not protective against CVD and even that they should be avoided due to various side effects [6, 7]. These reports have resulted in a reduction in compliance with secondary prevention therapy among Turkish patients diagnosed with CAD and a failure to reach therapeutic targets in many patients using statins [8].

The aim of this study was to determine what proportion of patients being followed for CAD were using statins and at what doses, to identify the reasons noncompliant patients were not using statins, and to determine the mean LDL-C levels of these patients. We also aimed to investigate how aware patients were that, besides lowering cholesterol, statins can slow and reverse the progression of CAD through their pleiotropic effects, and we wanted to determine to what degree information provided by medical professionals and negative media coverage influenced patients' rates of treatment compliance.

## 2. Materials and Methods

Patients diagnosed with CAD by angiography in the cardiology outpatient clinics in three centers in our province between October 2016 and January 2017 were included in the study. In our study, coronary artery disease was defined as a stenosis of 50% or more in a coronary artery of 1.5 mm or more diameter shown via coronary angiography [9]. The researching physician recorded patients' demographic data, current medical treatment, most recent blood analysis results (lipid profile, creatinine, aspartate transaminase [AST], alanine transaminase [ALT]), and left ventricular ejection fraction (EF) from echocardiogram done within the previous 6 months. Hemogram analysis was done using a Beckman Coulter LH 780; lipid panel, liver function tests, and renal function tests were done using a Roche Cobas C501. 1). In our country, statins are prescribed to every patient with coronary artery disease at time of diagnosis unless there is contraindication. Our study included patients who started statin therapy when they were diagnosed with coronary artery; any patients with contraindications for the use of statins were excluded. In the 2013 ACC dyslipidemia guidelines, atorvastatin 40 and 80 mg and rosuvastatin 20 and 40 mg were defined as high-intensity statin therapy, while atorvastatin 10 and 20 mg, pravastatin 40 mg, and rosuvastatin 10 mg were defined as moderate-intensity statin therapy. The patients in the study were divided into 3 groups: those with no statin use, moderate-intensity statin use, and high-intensity statin use. Due to the lack of scientific evidence of the efficacy of the herbal products in coronary artery disease, these patients were included in the drug-free group. The patients were asked 5 questions to assess their knowledge about the use of statins in secondary prevention of CAD. Of the patients who presented to the cardiology outpatient clinics, we excluded patients who were under 18 years old, had hearing problems or advanced dementia or contraindication of statin use, or were not willing to participate in the study.

The study was approved by the local clinical research ethics committee (2016/313). All patients were fully informed about the study before being enrolled. Data were collected using a form prepared in line with the aims of the study. In the first section of the form, the examining physician recorded demographic data, details regarding currently used medications, and information about comorbidities. The second section of the form contained 5 questions concerning the medical treatment patients should be taking. This section was filled in by the patient.

The minimum patient number for the study to show significance was determined based on the results of power analysis. Normality of the data distributions was tested for each group using the Shapiro-Wilks test. Normally distributed parameters were expressed with the descriptive statistics mean and standard deviation; parameters not showing normal distribution were expressed as median and percentiles. Categorical data were expressed as number and percentage. Differences between pairs of groups were evaluated using the Mann–Whitney test when the parameter was not normally distributed. Differences between three groups were evaluated by analysis of variance (ANOVA) for parameters with normal distribution and Kruskal-Wallis test for parameters with nonnormal distribution. The Tukey test was used as a post hoc test. Relationships between categorical parameters were evaluated using chi-square analysis. SPSS version 16.0 statistical software was used for all statistical analyses. The chi-squared and Student's t-test were used for descriptive statistics. Level of significance was accepted as p≤0.05.

## 3. Results

The patients included in the study were divided into 3 groups, those with no statin use, moderate-intensity statin use, and high-intensity statin use. The patients' demographic characteristics, risk factors, and LDL-C levels are shown in Table 1. There were no significant differences in rates of statin use when patients were compared based on age, sex, creatinine levels, or presence of diabetes mellitus, hypertension, or heart failure. The rate of alcohol consumption and the rate of illiteracy were higher in the drug-free group than statin group** (p<0.001)**. The mean LDL-C levels of the high-intensity statin, moderate-intensity statin, and no statin groups were 78.7**±**28.1, 97.2**±**26.9, 126.6**±**26.3, respectively, and pairwise comparisons showed that the differences in LDL between groups were significant** (p<0.001) (Table 1)**. Eighteen percent of the patients had LDL-C levels below 70 mg/dL as recommended in the relevant guidelines. AST levels were highest in the high-dose statin group but were still within normal range.

**Figure 1:**
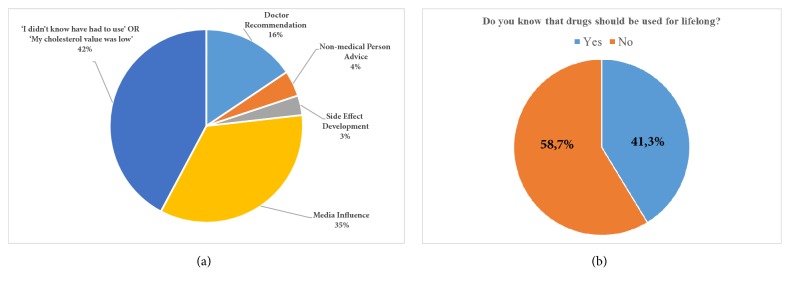
(a) Main reasons for not using medications stated by patients. (b) The rate of knowing that drugs should be used for lifelong.

When the** 217** patients who did not use a statin were asked why not,** 42**% responded by saying their cholesterol was not high or that they did not know they were supposed to renew their prescription once they ran out. Another** 35**% stated that they did not use a statin because they were influenced by news reports in the media claiming that cholesterol-lowering drugs were harmful. Another** 16**% stopped taking statins by doctor's recommendation, and** 3**% did not take statins because they developed side effects to the drug. Four percent of the patients stopped taking the drug as a result of a friend's (nonmedical person) advice** (Figure 1(a))**.

**Figure 2:**
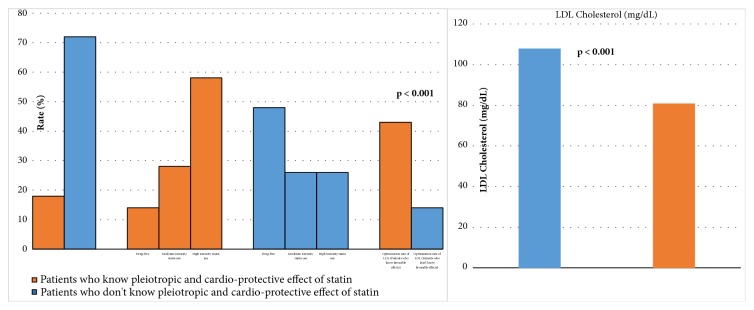
Differences of patients groups according to awareness of pleiotropic/cardioprotective effects of statins.

**Figure 3:**
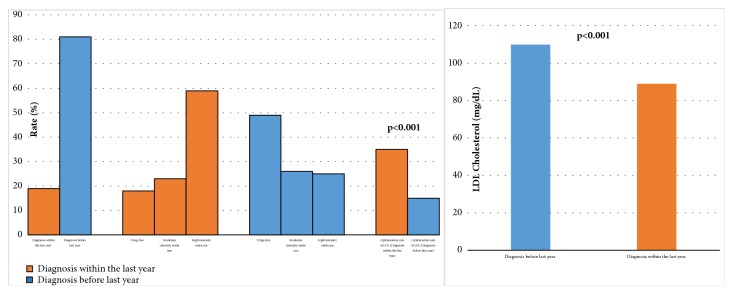
Differences of patients groups according to coronary artery disease diagnosis year.

When patients who were aware of the pleiotropic/cardioprotective effects of statins were compared with patients who were not, we found that the more knowledgeable patients had lower treatment noncompliance rate and mean LDL-C level and a higher rate of LDL-C level optimization** (Figure 2)**. Patients who were diagnosed with CAD in 2016 accounted for** 23**% of the study participants, and this group had lower statin noncompliance rate and mean LDL-C level and a higher rate of LDL-C level optimization** (Figure 3)**.

**Figure 4:**
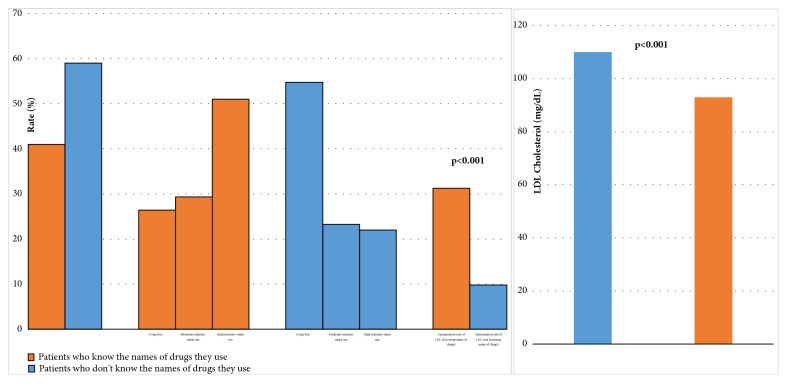
Differences of patients groups according to know the names of drugs.

Overall,** 41**% of the patients in the study knew the name of the drug they used. Patients who knew the name of the drug they used also had lower statin noncompliance rate and mean LDL-C level and a higher rate of LDL-C level optimization when compared with those who did not** (Figure 4)**. Due to the lack of scientific evidence of the efficacy of the herbal products in coronary artery disease, these patients were included in the drug-free group. The LDL-C levels of patients who used statin therapy for secondary prevention and those who used herbal products to lower their cholesterol were** 97.5 ± 27** and** 126.2 ± 24.9**, respectively** (p<0.001)**. When asked if they were aware that they needed to take the statin for the rest of their lives, only** 41.3**% indicated that they did** (Figure 1(b))**.

## 4. Discussion

In this study, we found that rates of statin use were low and LDL-C levels exceeded the values recommended in the ESC 2016 guidelines among diagnosed CAD patients being followed in cardiology outpatient clinics who should be receiving secondary hyperlipidemia prophylaxis. Less than a third (31.1%) of the patients being followed for CAD who should be using a statin for secondary prevention were taking high-intensity statin therapy. For 35% of the noncompliant patients, their reason for not taking statin therapy was negative news seen in print or visual media; 42% did not continue their therapy because they believed their cholesterol was not high or did not realize statins must be taken indefinitely. Side effects caused another 3% to stop taking statins, and 16% reported that their physician told them they did not need to continue taking a statin. Compared with patients diagnosed earlier, those who were diagnosed with CAD within the previous year were more likely to comply with statin therapy and had lower LDL-C levels. Only 16.9% of the patients in the study were aware of the cardioprotective pleiotropic effects of statins. Patients who knew of this property showed higher rates of statin use and lower LDL-C levels.

In a phase IV clinical trial evaluating the use of simvastatin for secondary prevention in CAD patients, simvastatin therapy resulted in a 35% reduction in LDL-C and a 34% reduction in major cardiac events [10]. In the REVERSAL [11], ASTEROID [12], and SATURN [13] studies of plaque regression, aggressive lipid-lowering therapy caused plaque regression parallel to the decrease in LDL-C and significantly reduced major cardiac events. The MIRACL study demonstrated that statin therapy can increase plaque stability, lower the incidence of acute coronary syndrome, and reduce recurrent coronary ischemia [14]. Plaque instability is related to a thin fibrous capsule and high macrophage content, and lowering the lipid content in coronary plaque formation increases the stability of the plaques. Statins enhance plaque stabilization by decreasing macrophage accumulation and cholesterol ester content in atheromatous plaques and increasing the volume of the collagen component. It has been shown with optical coherence tomography (OCT) that optimal lipid-lowering therapy after drug-eluting stent implantation lowers LDL-C level and protects against neointimal thickening [15]. Clinical studies and observations have shown that statins have other beneficial effects irrespective of their lipid-lowering effect. These are referred to as “pleiotropic effects” and include improving or restoring endothelial function, reducing oxidative stress and vascular inflammation, enhancing the stability of atherosclerotic plaques, and inhibiting thrombogenic reaction. Low compliance with statin therapy among CAD patients not only increases mortality and morbidity, but also significantly increases the burden on health care services due to preventable cardiac events.

The rates of statin use for secondary prevention and rates of achieving target LDL-C levels are quite low in Turkey and Europe [16–18]. In concordance with other studies, we found that 56.9% of CAD patients were using statins. The 2013 ACC/AHA Guideline on the Treatment of Blood Cholesterol to Reduce Atherosclerotic Cardiovascular Risk in Adults recommends initiating high-intensity statin therapy for CAD patients under 75 years old not using any statins and increasing therapy to high-intensity in patients on low- or moderate-intensity statin therapy [19]. In our study, patients on high-intensity statin therapy comprised 31.1% of the entire patient group and 54.7% of all patients using statins. We found that patients on high-intensity statin therapy were significantly more likely to have LDL-C values under 70 mg/dL. We believe that encouraging physicians to favor higher doses when prescribing statin-containing drugs will result in more favorable results in terms of cardiovascular outcome measures. In 2010, Yiğiner et al. reported that 23.7% of type 2 diabetes patients using statins for secondary protection reached target LDL levels [20]. The target LDL-C level in the present study was under 70 mg/dL, and 18.4% of the patients reached this target. The lower proportion of patients with target LDL-C levels in our study may be explained by the fact that previous studies used a target LDL-C level of 100 mg/dL. The mean LDL-C level of the patients in our study was 104.1 mg/dL, which is far from the 70 mg/dL limit specified in the 2016 ECS Guidelines for the Management of Dyslipidemias. This can be attributed to low compliance with statin therapy and insufficient prescription of high-intensity statin therapy as recommended in the guidelines.

In our study, we asked patients 5 questions to assess their level of knowledge about statin therapy. When patients with CVD was asked about the necessary duration of medical therapy, 41.3% knew that the condition required lifelong treatment, and these patients' LDL levels were lower than those who were not aware of that fact. Studies have demonstrated that the consistent use of statins has positive effects which reduce cardiovascular risk [21]. We believe that patients who know that CVD is a lifelong disease that requires lifelong treatment are more motivated and more compliant with medical therapy. We also observed that patients who were diagnosed with CAD within the last year were significantly more likely to take statin therapy and had significantly lower LDL-C levels when compared with patients who were diagnosed earlier. This may be because compliance with treatment is higher in the first year after CAD diagnosis and decreases in later years either because patients do not know statin therapy is a lifelong treatment, or because patients are more influenced over time by external factors such as the media. Emphasizing to CAD patients that statins must be used continuously should have a positive impact on the achievement of therapeutic targets. We believe that patients attend follow-up in the cardiology outpatient clinic more regularly in the first year after being diagnosed with coronary artery disease. In the following years, patients may not appear regularly for follow-up or may go to their family doctor or to clinics other than cardiology, such internal medicine, for prescriptions. Some patients stop attending follow-up altogether because they think their disease is completely healed. Statins were prescribed to all patients who presented to our clinic, both in the first year and in subsequent follow-up visits. However, we do not know how many patients attending other centers were given statin or high-intensity statin prescriptions. Furthermore, some patients who are prescribed statins do not adhere to treatment. We believe these factors explain the apparently higher rates of statin and high-intensity statin usage in the first year after diagnosis. Furthermore, reluctance of physicians in branches other than cardiology to prescribe high-intensity statin therapy may have contributed to more patients using lower intensity statin regimens. It may be beneficial for noncardiologists to stay informed regarding updated guidelines on prescribing high-intensity statin therapy for CAD patients.

In the present study, 41.3% of the patients knew the name of the drug they used. We believe that encouraging patients to learn the name of the medications they take may contribute to more effective treatment. We consider patients knowing the names of drugs they use to be an indicator that they are better informed about their therapy. This may be important in terms of awareness of which medications are used for which indication. This, in turn, may promote treatment adherence. However, higher treatment adherence in this group may also be associated with the higher sociocultural level of patients who know the name of the drugs they use.

Only 16.9% of the patients in our study were aware of the pleiotropic effects of statins and knew that they are prescribed not only to lower cholesterol, but also for their other cardioprotective effects. The patients who were aware of the pleiotropic effects of statin therapy had higher rates of statin use, lower LDL-C levels, and a higher proportion of LDL-C optimization when compared with patients who did not know of those effects** (Figure 2).**

Over a third (35%) of patients not using statins for secondary prevention stated that they had been influenced by visual or printed media reports that statin use was not beneficial or was even harmful. Previous studies conducted in Turkey also showed that the media has a negative impact on statin use. In a study on diabetic patients, Keskin et al. reported that 52.3% of those who stopped taking statin therapy did so because of news in the media [22]. Dinçer et al. also determined that negative media coverage was responsible for treatment noncompliance in 52.9% of patients attending outpatient clinics who should have been taking statin for secondary prevention but were not. Despite numerous studies demonstrating the beneficial effects of statin drugs in the secondary prevention of CAD and their recommendation in the both the ACC and ESC treatment guidelines, reports indicating that statins have no benefit and may even be harmful appear in the visual and printed media in Turkey. Kocas et al. found that statin usage was decreased during the period of increased number of news about statins in media [23]. Similar to this study Bezin et al. found that as a result of negative news about statin use in media this decreases statin compliance of patients. This decrease was more significant in patient with high risk population in terms of coronary artery disease and this situation was associated with higher mortality in this population [24]. As we observed in our study, this leads to patients with atherosclerotic heart disease to be deprived of the medication they need.

Medical treatment has a substantially positive effect on mortality and morbidity in patients with CVD. We believe that CAD patients are adequately informed about drug effects in daily practice, yet the results of our study show that the majority of these patients do not know enough about medications, especially statins. We believe that spending more time explaining and better emphasizing the mechanisms of action, reason for prescribing, and necessary treatment duration of the drugs that patients must use will result in greater compliance and improve patient care. In this way, patients may be less influenced by misinformation presented by the media. In addition, it would be beneficial for a competent medical staff member to follow patients' treatment compliance and, if necessary, do follow-up telephone visits in order to explain the effects of cardiovascular drugs.

## Figures and Tables

**Table 1:** Demographic, echocardiographic, and clinical features of coronary artery disease patients according to statin use.

	Drug-Free Group n=217	Moderate-Intensity Statin Group n=130	High- Intensity Dose Statin Group n=157	p
Age, *year*	62.1 ± 9.8	63.7 ± 8.9	62.5 ± 9.4	0.32
Gender, *male ***(n,%)**	148, %40.1	99, %26.8	122, %33.1	0.08^**a**^
DM, *present ***(n,%)**	85, %49.4	40, %23.3	47, %27.3	0.11^**a**^
HT, *present ***(n,%)**	127, %41.8	85, %28	92, %30.3	0.39^**a**^
Creatinine, *mg/dl*	0.88 [0.74-1.0]	0.9 [0.79-1.0]	0.89 [0.79-1.0]	0.18
AST, *U/L*	20 [16.5-24]	21 [17.7-24]	24 [19-32]	**<0.001**
LDL Cholesterol, *mg/dL *	126.6 ± 26.3	97.2 ± 26.9^**b**^	78.7 ± 28.1^**b**,**c**^	**<0.001**
Active Smoking, **(n,%)**	64, %55.2	20, %17.2	32, %27.6	**0.007** ^**a**^
Heart Failure, *present ***(n,%)**	40, %38.5	31, %29.8	33, %31.7	0.47^**a**^

	Drug-Free Group n=217	Moderate and High Intensity Statin Group n=287		p

Literacy, **(n,%)**	187 (%86.1)	278 (%96.8)		**<0.001** ^**a**^
Alcohol consumption, **(n,%)**	25 (%11.5)	8 (%2.7)		**<0.001** ^**a**^
LDL Cholesterol < 70 *mg/dL*	1 (%0.4)	93 (%32.4)		**<0.001** ^**d**^

***DM;*** *diabetes mellitus,* ***HT;*** *hypertension,* ***AST;*** *aspartate aminotransferase,* ***LDL;*** *low density lipoprotein.*

^a^p =x^2^ value; ^**b**^p<0.05 versus drug free group; ^**c**^p<0.05 versus low dose statin group; ^d^p=Fisher's extract test.

*Values are expressed as mean ± standard deviation or median (1st–3rd quartiles), as appropriate.*

*p value below* ***0.05*** *was considered significant and significant parameters were shown by* ***bold type.***

## Data Availability

The data used to support the findings of this study are available from the corresponding author upon request.
